# Atypical presentation of a middle age male with severe hypertriglyceridaemia: a case report

**DOI:** 10.1186/1752-1947-1-51

**Published:** 2007-07-14

**Authors:** Ali I Albahrani, Jannette Usher J, Eileen Marks, L Ranganath, Alan Shenkin

**Affiliations:** 1 Department of Clinical Biochemistry, St Mary's Hospital, Newport, Isle of Wight, PO30 5TG, UK; 2 Royal Liverpool University Hospital, Duncan Building, 4th floor, Liverpool, L69 3GA, UK

## Abstract

**Background:**

Severe hypertriglyceridaemia (HTG) is uncommon but most prevalent in subjects with type 2 diabetes mellitus (T2DM) and excess ethanol intake.

**Case presentation:**

We describe a case of a middle age male (53 y) presenting to the emergency room with acute atypical central chest pain and severe HTG in the absence of evidence of overt ischaemic heart disease (IHD). Admission ECG and EET (exercise tolerance test) were negative for reversible ischaemic changes. His admission glucose was 12.2 mmol/l, triglycerides (TG) were 103 mmol/l, total cholesterol 37 mmol/l. Cardiac Troponin T could not be measured on three occasions but CK MB mass was normal at 3 μg/l. The patient was started on Bezafibrate 400 mg OD, Simvastatin 20 mg nocte, Omacor (Omega-3 fish oil) 1 gm bd and Metformin 500 mg tds. Four weeks after admission, lipid and liver profiles showed remarkable improvement, TG 2.9 mmol/l, Tchol 6.3 mmol/l and HDLc 1.5 mmol/l, ALAT and GGT were normal.

**Conclusion:**

A case report of severe hypertriglyceridaemia with atypical presentation demonstrate the role of combined lipid modifying agents in lowering triglycerides and cholesterol as well as improving liver enzymes.

## Background

Severe hypertriglyceridemia (HTG) is an uncommon metabolic disorder. Prevalence of severe HTG, defined as triglycerides (TG) greater than 22 mmol/l, is estimated to be 1.8 cases per 10,000 adult Caucasians [1]. It is exacerbated by uncontrolled diabetes mellitus, obesity, excess ethanol intake and sedentary habits, all of which are more prevalent in industrialized societies than in developing nations.

TG are synthesized in the liver and intestine and packaged into lipoproteins. Chylomicron is synthesized in the intestine, and very-low-density-lipoprotein (VLDL) is synthesized in the liver. Chylomicron and VLDL normally undergo rapid metabolism via the action of lipoprotein lipase (LPL), hepatic lipase (HL), and cholesterol ester transfer protein (CETP). During catabolism, any disturbance that causes increased synthesis of chylomicron and/or VLDL or decreased metabolic breakdown will cause elevations in TG levels [1]. That disturbance may be as common as dietary indiscretion or as unusual as a genetic mutation of an enzyme in the lipid metabolism pathway. Two rare genetic causes of severe HTG are LPL deficiency and apolipoprotein (apo) C-II deficiency lead to TG elevations that are astonishingly high. A common genetic form called familial combined hyperlipidaemia (FCHL) characterised by multiple lipoprotein phenotypes and strongly associated with premature cardiovascular disease. The prevalence of FCHL is 1/500 in adult caucasians, the genetic of FCHL is not yet clearly defined [2]. The commonest presentations of severe HTG are recurrent episodes of severe abdominal pain (acute pancreatitis) and eruptive xanthomata (which occur when TG-rich lipoproteins are taken up by skin macrophages in discrete locations usually involving the back and proximal extremities) to the dermatologists.

## Case presentation

Here we describe a case of 53-year-old white male admitted to the emergency room with acute atypical central chest pain of 8 hours duration. The chest pain was mainly at rest; was non-radiating and was not associated with sweating, palpitation, nausea or vomiting. He reported three similar episodes during the previous week. He denied any alteration in his bowel habits or other gastro-intestinal symptoms. His only medical problem was hypertension that was diagnosed 6 months ago and he was commenced on Bendrofluazide but he admitted being non-complaint with his medication. He smoked 20 cigarettes/day and drank 6–8 units/day. His father and five of his paternal uncles died from IHD in their early fifties. His mother died at the age of 57 from carcinoma of the cervix and he has one living sister. He walked up to 5 miles a day without any chest discomfort prior to this admission.

On examination, he was tachycardiac at 103 b/min but in sinus rhythm, blood pressure 167/129 mmHg, oxygen saturation 97% at room air, respiratory rate 20/min and temperature was normal at 36.7°C. BMI was 27.6. Cardiovascular, respiratory and abdominal examinations were unremarkable. Fundoscopic examination revealed lipaemia retinalis. A 12 lead admission ECG was negative for ischaemic changes apart for peaked T waves. Chest X-ray did not revealed any abnormality. Troponin T could not be measured on three occasions due to lipaemia (hypertriglyceridaemia) but CK-MB was normal 3 μg/l. Exercise tolerance test on the next morning was negative for any reversible ischaemic changes. Admission lipid profile revealed a markedly raised TG at 103 mmol/l. Next morning repeats lipid profile; TG and total cholesterol (Tchol) were 57 mmol/l and 32 mmol/l, respectively. HDL cholesterol was unmeasurable with urea 4.5 mmol/l, creatinine 86 μmol/l, total protein 67 g/l, albumin 39 g/l and urine albumin/creatinine ratio 9.7. Fasting glucose and HBA1_c _were normal at 4.5 mmol/l and 5.8%, respectively. Liver function test revealed a moderately raised ALT and GGT at 60 U/l and 730 U/l, respectively. Serum amylase was normal at 51 U/l.

The Initial impression was a case of acute coronary syndrome and familial combined hyperlipidaemia. The patient was commenced on anti-angina medications, glycerine trinitrate (GTN) and β-blocker (Bisprolol) and for his hyperlipidaemia he was commenced on Bezafibarate 400 mg od. Five days post admission the pain improved to a great extent other than mild discomfort and a repeat lipid profile revealed a TG 13.7 mmol/l and Tchol 15.3 mmol/l. For that he was commenced on Simvastatin 20 mg nocte, Omacor 1 gm bd, and Metformin 500 mg tds in addition to the Bezafibrate. An urgent dietary review was arranged, with advice to reduce his fat intake to <10% of total calories intake. Two weeks after admission, lipid profile improved with TG and Tchol at 5.0 mmol/l and 8.3 mmol/l, respectively, HDL cholesterol was at 1.5 mmol/l. Four weeks from the acute presentation, he was reviewed in the lipid clinic with TG 2.9 mmol/l, Tchol 6.3 mmol/l and HDL cholesterol at 1.4 mmol/l. Liver profile improved with normal ALT at 30 U/l and mildly raised GGT at 60 U/l. Apolipoprotein E genotyping was apoE3/E4. The patient's chest pain and exercise tolerance improved to a great extent (he started regular exercise and he cutdown his ethanol intake to 2 units/day and smoking to 2–3 cigarettes/day). The cardiac team has reviewed the patient, his anti-angina medication was discontinued and he was discharged from the cardiac clinic.

This is a middle age male with most probably FCHL exacerbated by excess ethanol intake, being over-weight and insulin resistant. FCHL is caused by hepatic over-production of VLDL, either with or without impaired clearance of TG-rich lipoproteins from plasma. In this group of patients there is a compromise of chylomicron clearance, perhaps due to competition for the same common pathway of lipid hydrolysis in the vascular compartment [2]. The diagnosis of FCHL in this patient is based on presence of moderately to markedly raised Tchol. The familial nature of our patient hyperlipidaemia is illustrated by the fact that his father and five of his paternal uncles have died from IHD in their early fifties. Despite the fact that our patient did not reveal any evidence of ischaemic changes on ECG and ETT, nonetheless, these tools do not exclude occult coronary atherosclerosis. Besides subjects with FCHL are at high risk of future IHD [2]. The possibility of acute pancreatitis has been rule out based on clinical presentation; normal C.T abdomen and serum amylase and improved pain pattern few days post admission.

Our patient presentation provides examples of the various features of severe HTG, which included milky appearance of the retinal vasculature, the normal deep-blue colour of the veins and bright-red colour of the arteries blend into a pink colour, making each difficult to distinguish, a condition called lipaemia retinalis. In addition, our patient presented with atypical chest pain in the absence of ischaemic changes on ECG and ETT. It is thought that the aggregation of VLLD and chylomicron can obstruct the capillary bed, resulting in tissue ischaemia [1]. Reviewing the literature, we were able to find two case reports of severe HTG presenting with acute chest pain in the absence of evidence of IHD, one patient was known to have T2DM and the other patient was a pregnant lady [3,4]. The relationship between TG and cardiovascular is less clear compared to cholesterol. However, there are a number of postulated mechanisms that have linked raised TG with IHD. These include retention of chylomicron and VLDL remnants, small dense LDL, low HDL and increased coagulability of the plasma [1]. There have been multiple conflicting studies regarding the role of triglycerides and the development of IHD. HTG is clearly associated with IHD in univariate analysis [1]. However, many multivariate studies have shown that its risk is markedly attenuated after adjustment for other IHD risk factors, namely low HDL and increased small, dense LDL particles. A recent review of the literature concluded that treating isolated HTG does not prevent coronary events [5]. On the other hand; there have been many other studies that have shown HTG to be an independent risk factor for IHD even after adjustment for HDL and LDL [6-8].

Despite the controversy that exist between hypertriglyceridaemia and IHD, severe hypertriglyceridaemia is well established cause of pancreatitis, therefore, it is imperative to manage subjects with severe hypertriglyceridaemia in order to avoid pancreatic damage [9].

Our patient was commenced on a Fibrate which is a specific transcription factors belonging to the nuclear hormone receptor superfamily, termed peroxisome proliferator-activated receptors (PPARs). Fibrates work by lowering hepatic apoC-III production and increasing lipoprotein lipase that will results in a decrease in VLDL production [10]. Our patient's TG improved over a period of five days, nonetheless, there was reversed increase in the ratio of Tchol to TG. Fibrates are known to be associated with paradoxical increase in LDL cholesterol in certain types of hyperlipidaemia especially type IV hyperlipidaemia [10]. For that the patient was commenced on Simvastatin (3-Hydroxy-3 methylglutaryl CoA reductase inhibitors) and Metformin (*N*^1^,*N1*-dimethylbiguanide), and Omacor (Omega-3 fatty acids). Combinations of lipid modifying agents have resulted in significant improvement in the TG. At this stage the patient was advised to continue on the current medications due to the strong family history of IHD and protection against pancreatic damage that could predispose him to diabetes. Moreover, the patient was adviced about the importance of lifestyle changes, weight reduction, ethanol and smoking cessation. The remarkable improvement in the triglycerides level over the proceeding days post admission has precludes the need for a more invasive approach in managing this patient severe hypertriglyceridaemia by plasmapheresis.

This case report illustrates the importance of lipid lowering agents on clearing fat deposit (steatosis) from the liver. Despite the fact that more than two lipid-lowering agents which some time can cause liver dysfunction were used. There were well tolerated by the patient, and there was a paradoxical improvement in his liver enzymes, both ALT and GGT being normalised within the reference range over a period of four weeks. Therefore, an understanding of the pathogenesis and natural history of this metabolic condition will help to identify the subset of patients with fatty liver that could benefit from medical therapy, despite the associated risk of hepat-toxicity with Statin and Fibrates. Small studies on the use of Metformin and Thiazolidinediones (insulin sensitizers) and Fibrates in animal models and in subjects with T2DM with fatty liver have also resulted in normalization of ALT.

In some of these studies Metformin and Fibrates reversed hepatomegaly and steatosis on liver biopsy. [11]. The patient was recently reviewed in the lipid clinic, his general well-being and exercise threshold have improved to a great extent, the pain almost disappear completely with very un-occasional chest discomfort, the cardiac team have discharged the patient from the cardiac clinic based on two ETTs.

## Conclusion

This is an interesting report of severe hypertriglyceridaemia with an atypical presentation, which demonstrates the role of combined lipid modifying agents in lowering triglycerides and cholesterol as well as reciprocal improvements in liver enzymes.

Method: Plasma triglycerides, total cholesterol, ALT, GGT, glucose, creatinine, urea, sodium, potassium and total ALP were measured using the standard methods on Roche Modular analysers (Lewis. UK).

HDL cholesterol measured using PEG-modified enzymes and dextran sulphate. Within and between batch precision at 1.0 mmol/l were 0.9% CV (Human serum) and 1.85% CV (Human serum), respectively.

HBA1_c _was measured using high performance liquid Chromatography (HPLC). Within and between batch precision at 4.54%, 7.22% and 12.87% were 1.1, 1.3, and 1.0, and 1.1, 1, and 0.5, respectively.

Apolipoprotein E (apoE) was measured using polymerase chain reaction (PCR).

BMI was calculated as weight (kg) divided by height (m^2^) and used as an index of adiposity.

## Competing interests

The author(s) declare that they have no competing interests.

## Authors' contributions

AB was involved in the management of the patient as well as writing the case reports. UJ has carried out the ApoE genotyping. LR was involved in the management of the patients. EM and AS has been involved in the correction of the manuscript as well as general supervision. All authors read and approved the final manuscript.
